# Political polarization: a curse of knowledge?

**DOI:** 10.3389/fpsyg.2023.1200627

**Published:** 2023-07-12

**Authors:** Peter Beattie, Marguerite Beattie

**Affiliations:** ^1^MGPE Programme, The Chinese University of Hong Kong, Shatin, Hong Kong SAR, China; ^2^Faculty of Social Sciences, Faculty of Educational Sciences, University of Helsinki, Helsinki, Finland

**Keywords:** curse of knowledge, cognitive bias, debiasing, political polarization, political epistemology

## Abstract

**Purpose:**

Could the curse of knowledge influence how antagonized we are towards political outgroups? Do we assume others know what we know but still disagree with us? This research investigates how the curse of knowledge may affect us politically, i.e., be a cause of political polarization.

**Background:**

Research on the curse of knowledge has shown that even when people are incentivized to act as if others do not know what they know, they are still influenced by the knowledge they have.

**Methods:**

This study consists of five studies consisting of both experimental and non-experimental and within- and between-subjects survey designs. Each study collected samples of 152–1,048.

**Results:**

Partisans on both sides overestimate the extent to which stories from their news sources were familiar to contrapartisans. Introducing novel, unknown facts to support their political opinion made participants rate political outgroup members more negatively. In an experimental design, there was no difference in judging an opponent who did not know the same issue-relevant facts and someone who did know the same facts. However, when asked to compare those who know to those who do not, participants judged those who do not know more favorably, and their ratings of all issue-opponents were closer to those issue-opponents who shared the same knowledge. In a debiasing experiment, those who received an epistemological treatment judged someone who disagreed more favorably.

**Conclusion:**

This research provides evidence that the curse of knowledge may be a contributing cause of affective political polarization.

## 1. Introduction

“*[T]he opponent presents himself as the man who says, evil be thou my good. … [H]e who denies either my moral judgments or my version of the facts, is to me perverse, alien, dangerous. How shall I account for him? The opponent has always to be explained, and the last explanation that we ever look for is that he sees a different set of facts*.”

Walter Lippman, *Public Opinion.*

The “curse of knowledge” fundamentally consists of an impaired ability to imagine the reasoning of others who do not share one’s knowledge. This is caused by an implicit presumption that one’s own knowledge is shared by others, a presumption that is largely impervious to evidence that others do *not* share this knowledge (Birch and Bloom, 2007; Dębska and Komorowska, 2013; *cf.* Ryskin and Brown-Schmidt, 2014). This bias is likely produced by a combination of fluency misattribution (mistaking the fluency or ease with which information comes to mind, with how widely shared that information is) and a failure of inhibitory control (inhibiting one’s own knowledge while estimating what others know; Birch et al., 2017). Drayton and Santos (2018) found evidence that non-human primates do not exhibit curse-of-knowledge effects, suggesting it is unique to the theory of mind humans have evolved. As such, it may have been evolutionarily adaptive for its efficiency; as hyper-cooperative or eusocial animals living in small groups for most of our history (Wilson, 2012), we inhabit “knowledge communities” wherein we take the knowledge held by members of our own community, or even the internet, to be the same as our own knowledge (Fisher et al., 2015; Sloman and Rabb, 2016; Rabb et al., 2019). The curse of knowledge is essentially the inverse: assuming our knowledge is the same as that held by members of our community.

The term “curse of knowledge” was first coined in an economic experiment (Camerer et al., 1989). The study tested the uncontroversial assumption that in economic situations featuring asymmetric information, marketplace participants with more information than others would be able to accurately predict the judgments of participants without this information — and profit from the information asymmetry. What the experimenters found, however, is that even with real money to be gained, their subjects had difficulty predicting the judgments of people *lacking* the information they had. Although they had been instructed that other marketplace participants lacked certain key information they had been given, subjects made investment decisions influenced by an apparently unconscious assumption that all other marketplace participants also knew what they knew — and lost money as a result. Rather than serving as an advantage, the knowledge unique only to the subjects operated as a curse.

Paradoxically, the authors noted that in economic settings, this curse of knowledge may actually *increase* social welfare (Camerer et al., 1989, p. 1245). Information asymmetries are conventionally thought to produce economic inefficiencies, as when a used car dealer overcharges for a “lemon,” because only the seller knows hidden defects of which the buyer is ignorant. In such economic settings, the individual curse of knowledge might be a blessing for society, by making information asymmetries invisible to, and less likely to be exploited by, the party with more information. Hence in these economic settings, the curse of knowledge may be an example of psychological vice producing public virtue.

Yet in political settings, there is reason to expect the curse of knowledge to *reduce* social welfare. The curse of knowledge may describe a psychological “default” setting, or an innate theory of mind we use to understand others, in which everything we know is considered common knowledge, shared by all of our interlocutors (Nickerson, 1999). They are then treated accordingly, as if they knew the same information we have learned. This would make it more difficult to accurately understand the thinking of others who do *not* share knowledge that supports our political opinions. (Hence the *curse* of knowledge: it can make our understanding of other people’s thinking worse than when we are ignorant of particular knowledge).

How might we understand the thinking of political opponents, when the curse of knowledge makes us implicitly assume that they have learned the same knowledge we have learned, i.e., the information that has shaped our opinion of an issue? For example, imagine two U.S. Americans in 2003 with opposing views on the invasion of Iraq. One may have supported the invasion on account of the following knowledge: claims of Iraqi weapons of mass destruction and links with al Qaeda, and past atrocities committed by the Iraqi government under Saddam Hussein. The other may have opposed the invasion, on account of knowing the same information but also having additional knowledge: Hussein’s history of antipathy toward Islamic fundamentalism, UN weapons inspectors finding no WMD, and global public opinion disfavoring an invasion. If the supporter is affected by the curse of knowledge – implicitly assuming that everyone knows just what they themselves know (plus *unknown* unknowns) – what could possibly explain opposition to the war, other than opponents being careless about an existential threat at best, or “Saddam lovers” at worst? If the opponent is affected by the curse of knowledge, what could possibly explain support for the war — since their unique knowledge shows claims of WMD and al Qaeda links to be implausible — other than supporters being warmongering imperialists, motivated by the desire to control Iraq’s oil?

If the curse of knowledge operates in political thinking, it may compound or exacerbate (affective) political polarization. As we implicitly assume that our political opponents know all of the facts that we know — knowledge which has helped shape our political opinions in the first place — then we may judge our opponents more harshly. That is, if we presume that others share the knowledge that has shaped our opinions, and made such opinions appear self-evidently correct to us, then our political opponents may take on a malevolent character. They are assumed to have all of the knowledge we had to arrive at the correct (our) conclusion, yet they persist in taking the wrong position; like Milton’s Satan, it may seem that they have made evil their good.

This paper reports a series of studies testing whether the curse of knowledge is evident in political cognition. The results suggest that the curse of knowledge may be a contributing cause of political polarization, one of the heretofore overlooked psychological factors (Eibach, 2021) operating alongside institutional causes (Iyengar et al., 2019; Wilson et al., 2020).

### 1.1. Studies of the curse of knowledge

While the curse of knowledge (hereafter, CoK) has not yet been studied as it relates to and affects politics, it has been studied in a variety of other contexts. As discussed earlier, the curse of knowledge has been shown to apply in economic contexts, inhibiting marketplace actors’ ability to profit from predicting the decisions of those who lack the same information (Camerer et al., 1989; Keysar et al., 1995; Loewenstein et al., 2006). The CoK can negatively affect lawyers, who may overestimate what jurors know about memory research relevant to eyewitness testimony, to the detriment of their clients (Terrell, 2014). It can impact criminal investigators and the accused alike, both of whom may overestimate what the other party knows about a crime (Granhag and Hartwig, 2008). It affects doctors, whose communications with patients can be made less effective by the CoK inflating doctors’ estimates of patients’ medically relevant knowledge (Howard, 2019; Lourenco and Baird, 2020). It affects businesspeople, who may overestimate the level of knowledge widely held within a firm about that firm’s organizational structure, impairing intra-firm coordination (Heath and Staudenmayer, 2000). Accountants and financial regulators may suffer CoK effects by overestimating knowledge relevant to predicting outcomes (Kennedy, 1995). Safety inspectors can suffer CoK effects, by assuming that site supervisors already know best practices they in fact do not (King, 2019). The CoK can also affect writers, making it more difficult to imagine their audience’s ignorance of plot points they are intimately familiar with (Tobin, 2009), and impede communication in general by making ambiguous statements seem unambiguous to the speaker (Tobin, 2014).

The CoK is related to several other psychological biases, and was itself inspired by research on hindsight bias. Fischhoff (1975) demonstrated that we are influenced by outcome knowledge in our predictions of the likelihood of different outcome possibilities, both when we are placing ourselves in the shoes of our past ignorant selves, or the shoes of ignorant others. The CoK is also related to egocentrism; though whereas egocentrism is a difficulty in understanding perspectives other than one’s own, the CoK is a specific difficulty in understanding a *less informed* perspective, not one that is better informed (Birch, 2005; Ghrear et al., 2016). Whereas egocentric bias weakens over development, with adults better able than children to inhibit their initial egocentric thinking (Epley et al., 2004), in some contexts adults exhibit greater CoK effects than children (Mitchell et al., 1996). The CoK also exhibits similarities to the false consensus effect, an overestimation of the extent to which others share our perspective on an issue (Spaulding, 2016), and pluralistic ignorance, an overestimation of the extent to which others do not share our cognition or behavior (Sargent and Newman, 2021).

Past research indicates that the CoK is persistent and difficult to eliminate. In economic contexts, monetary incentives and repeated iterations of predicting less-informed market participants’ decisions reduced CoK effects, but only by half (Camerer et al., 1989). Higher education is associated with reduced CoK bias, but explicit instructions to focus attention on others’ knowledge did not reduce CoK effects (Damen et al., 2018). (However, greater knowledge may actually worsen CoK effects, by hiding from one’s view the areas in which one is, and others are, ignorant; Son and Kornell, 2010). Higher perspective-taking ability is also associated with reduced CoK effects, but instructing subjects to take another’s perspective was not associated with lessening CoK bias (Ryskin and Brown-Schmidt, 2014; Damen et al., 2020).

We were unable to find any studies of the curse of knowledge as it relates to political cognition. Without existing research as a guide, one possibility is that the CoK has little or no effect on political thinking. Politics being an essentially allocentric domain, thinking about politics may involve greater focus on what others know and do not know, thereby overcoming the bias. Another possibility is that the CoK, by implicitly ascribing one’s own knowledge to others, is a form of intellectual humility (Hannon, 2020). By reducing intellectual arrogance, this form of unconscious humility may tend toward reducing political polarization.

The possibility we thought most likely is that the CoK exacerbates affective political polarization, by masking the differences in knowledge that led to the formation of opposing opinions. The essence of the phenomenon – in Gomroki et al.’s (2023, p. 354) formulation, “When one interacts with others, one unknowingly imagines that others have the same intellectual background to understand the subject” – in the context of political disagreements, would seem to result in more negative appraisals of contrapartisans. This builds on the original definition of the CoK as an inability to inhibit one’s own knowledge when imagining the thinking of others who do not share the same knowledge, shifting focus to its practical, real-world implication: that we act as if unconsciously assuming that others share the same information. This is similar to accounts of “naive realism,” the result of psychological biases in which our inability to grasp that others have different knowledge informing their political opinions leads us to assume the worst about them (Ross and Ward, 1996; Friedman, 2020). Naive realism consists of the assumption that we see reality objectively, and our opinions about it are formed through an unbiased and unmediated apprehension of “the” facts. The naive realist assumes that others also see reality objectively, and will arrive at the same opinions as themselves. To explain why some people nonetheless disagree with their opinions, the naive realist has three explanatory options: the opponent may (1) be biased by ideology or self-interest, (2) be lazy, irrational, or unwilling to follow “the” evidence to its logical conclusions, or (3) not know the same information (Ross and Ward, 1996, 110–111). If the CoK affects political cognition, this third option is less likely to be taken under consideration; and the remaining options all place one’s political opponents in a negative light.

People often attribute negative motives to others, committing the worst-motive fallacy (Reeder et al., 2005). (Hence Hanlon’s razor: “never attribute to malice that which is adequately explained by stupidity” — in which “stupidity” should be replaced with “ignorance”). We expected that the CoK may contribute to political polarization, by obscuring the (highly likely) possibility that one’s political opponents have arrived at contrary opinions because they do *not know* the same information that has shaped one’s own opinion, and *do* know different information that has shaped their opinion. With this explanation occluded, and relevant information implicitly assumed to be universally shared, one’s political opponents take on a malicious hue. For them to have arrived at an opposing opinion, after considering the same information, they must have opposing values, be “ideological” or biased by self-interest, or simply be lazy, unintelligent, or irrational. In other words, *because* people are imputing knowledge to people who do not have it, they may judge them more harshly. Lastly, if the CoK is part of the causal story behind political polarization, how might CoK effects be reduced in political contexts?

### 1.2. The present research

If the CoK exacerbates political polarization, the first place to look would be in the news media, the source of the basic informational building blocks that are used to form political opinions. Our first study asked partisans in Hong Kong and the U.S. about recent news stories, inquiring who was likely to know of the event or phenomenon described in the story. In this way, it lays out the direct or foundational evidence for the CoK applying in the political realm. Finding evidence of overestimating knowledge, our following studies measure what effect the CoK may have on affective polarization (and by investigating CoK effects on polarization – from overestimating knowledge to more negative feelings toward opponents due to their overestimated knowledge – providing further, indirect evidence of the CoK operating in political cognition). In other words, the first study investigates whether partisans think opponents know about partisan news stories more than they do, while the subsequent studies measure how this over-imputation of knowledge may affect feelings toward political opponents, and how this could be debiased. Our second study investigated whether learning novel information about a political issue would lead to more negative attitudes toward one’s opponents on that issue. Our third study sought to uncover whether partisans judge political opponents more favorably if they are told that they do not know the same issue-relevant facts. Finding no evidence that providing information on others’ opinion-relevant knowledge or ignorance affects personal judgments, in Study 4 we asked participants to list their own most important political issue and three facts about it, and then to judge those who disagree with them on the issue — both in general, and separately for opponents who knew and did not know the factual information supporting the participants’ position. This more direct method of focusing attention on knowledge gaps was associated with a moderation in judgments of less-informed others. In a final study, we attempted to debias potential CoK effects, testing a treatment condition comprising instructions to consider political opponents’ lack of knowledge and how that may influence their opinion on the issue. Our overarching research question is: Does the curse of knowledge, the overestimation of knowledge shared in common, exacerbate political polarization, leading to harsher judgments of opponents (since “they should know better”)? Table 1 presents the specific research questions.

**Table 1:** Studies in the current research and their respective research questions.

Study	Research questions
1	Do partisans overestimate the extent to which their political opponents know news stories/facts featured in their preferred media outlets?
2	If partisans gain new issue-relevant information – and are told that others are ignorant of it – do they nonetheless judge opponents on the issue more harshly?
3	If partisans are asked to judge one of two political opponents – the only difference between them being whether the opponent knows or does not know the same issue-related facts – does this information about a knowledge gap lead to less harsh judgments for those who do not share the same knowledge?
4	If partisans are asked to judge both political opponents who know and do not know the same issue-relevant facts, do they rate those who do not know the same facts less harshly? Is their rating of those who know the same facts closer to their rating of political opponents in general?
5	If partisans receive a simple political epistemology explanation of why people may disagree, do they judge political opponents less harshly?

In the following studies, we report sample size determination, data exclusion, measures, and manipulations where relevant. All data and research materials, including the surveys, are available on the OSF: https://osf.io/yc3tf/?view_only=525a070de6a74410aaa445f3f97cbbec. In addition, for both the sake of transparency and to inform future research, we wrote an appendix about the development of our studies and the lessons we learned, which can be found from the above link as well. Randomization for all experimental conditions was performed by Qualtrics, ethics approval was received from the relevant institution, and the studies’ designs and analyses were not pre-registered. All U.S. samples were collected via Prolific among self-identified Republicans and Democrats. Besides over-representing political partisans by design, these samples contained fewer ethnic minorities, older people, men, and those with lower levels of educational attainment than the national average; median income was comparable to the nation median.

## 2. Study 1

During the Hong Kong protests of 2019, one of the authors realized what should have been apparent beforehand: having added people from both sides of Hong Kong’s political divide (“yellow,” or pro-democracy, and “blue,” or pro-establishment) to his social media platforms, he began to notice that the two sets of partisans shared and commented on news stories covering entirely different events. To test whether partisans in Hong Kong were overestimating the extent to which news stories they found important were known outside of their partisan group, several questions were added to an unrelated study, and this formed the basis for a broader research proposal. This design was later adapted to the U.S. context in 2022, to test whether U.S. partisans overestimate knowledge of news stories important to their partisan group. We expected to find overestimation of knowledge shared across political divides: partisans claiming knowledge of their-side news stories at higher rates than contrapartisans, and partisans considering their-side stories to be “common knowledge” at higher rates than contrapartisans.

### 2.1. Method

#### 2.1.1. Participants and design

In Hong Kong, participants were recruited via handing out flyers at pro-democracy and pro-establishment protests, for a total of 449 participants (239 women, 49 pro-establishment, *M*_age_ 30.59, SD_age_ 12.73). During this period, pro-establishment protests were less frequent and less attended than pro-democracy protests, resulting in the lower sample size for pro-establishment respondents. In the U.S., participants were recruited via Prolific among self-identified Republicans and Democrats, for a total of 201, due to uncertainty about whether the large effect sizes from the Hong Kong study would be found in a relatively less polarized context (98 women, 103 Democrats, *M*_age_ 41.23, SD_age_ 14.64). The studies were designed to present recent news stories in partisan media outlets on both sides, asking participants to identify whether they heard of the story, and whether they believed that co-partisans and/or contrapartisans had also heard of it.

#### 2.1.2. Procedure and materials

In Hong Kong, participants responded to a longer survey on political opinions, with these questions about news stories included. In the U.S., the questions about news stories comprised the survey, plus demographic questions. In Hong Kong and the U.S., recent (late 2019 in Hong Kong, early 2022 in the U.S.) news stories were selected from media outlets favored by the pro-democracy and pro-establishment, or Democratic and Republican, partisan groups, respectively. The Hong Kong stories were selected from among those popular on social media from established media outlets, and the U.S. stories were selected from transcripts of the popular Rachel Maddow (for Democratic stories) and Tucker Carlson (for Republican stories) shows, excluding stories that were covered on both programs. (As the most popular cable opinion shows for U.S. partisans at the time, we assumed that they would cover stories of particular interest to their partisan audiences, and that these stories would be covered by other outlets of the same partisan leaning.) An example of a “pro-establishment” story is “A police officer was burned by a Molotov cocktail thrown by protesters,” and an example of a “pro-democracy” story is “A leader of the Junior Police Officers’ Association used the word ‘cockroaches’ to describe protesters.” An example of a Republican-media story is “In September [2021], Chicago experienced its deadliest month since 1992, reporting 89 homicides for the month,” and an example of a Democrat-media story is “Interviews with former Trump administration staffers and associates revealed that the former president often violated the Presidential Records Act by destroying government documents.”

#### 2.1.3. Measures

Participants were presented with a sentence summarizing the news stories. They asked who they thought knew of the story, from “I do not recall ever hearing about this, or I do not think this happened” to three options starting with “I heard of it…” and ending in a progressively larger audience of others with the same knowledge: from neither partisan ingroup nor outgroup members (“…but I think most other people do not know about it”) to only the partisan ingroup (“…but I do not think many [of the opposing party] know about it”) to both partisan ingroup and outgroup members (“…and I think almost everyone knows it – it’s common knowledge”). This provided story-aware participants epistemically sophisticated options (they heard of it, but most others may not have, or only co-partisans may have heard of it via their similar media diets), and an option representing the curse of knowledge (all others, including contrapartisans, assumed to share the individual’s own knowledge). To minimize survey length, whether participants had *not* heard of the story or whether they believed it to be untrue were collapsed into the first option; the remaining options entailed knowledge of the story and belief that it was real.

### 2.2. Results and discussion

In Hong Kong, our pro-establishment respondents answered that they had heard of the pro-establishment stories 95.4% of the time, but our pro-democracy respondents answered that they had heard of the pro-establishment stories 71.1% of the time, *t*(449) = 8.3, *p* < 0.001, *d* = 1.26, 95% CI [0.96, 1.55]. Similarly, our pro-democracy respondents answered that they had heard of the pro-democracy stories 94% of the time, but our pro-establishment respondents answered that they had heard of the pro-democracy stories 73.2% of the time, *t*(449) = 11.5, *p* < 0.001, *d* = −1.75, 95% CI [−2.04, −1.45]. Meanwhile, pro-democracy respondents answered that the pro-democracy stories were “common knowledge” 71.3% of the time versus 38.8% for pro-establishment respondents, *t*(449) = 8.3, *p* < 0.001, *d* = −1.25, 95% CI [−1.55, −0.95], and pro-establishment respondents answered that the pro-establishment stories were “common knowledge” 69.1% of the time versus 29% for pro-democracy respondents, *t*(449) = 10.6, *p* < 0.001, *d* = 1.60, 95% CI [1.30, 1.89]. These contrast with the percentages selecting the more epistemically sophisticated answer (“I heard of it, and I’m pretty sure most people *on my side* have heard of it too”), which was selected 3.7% of the time by pro-democracy respondents for their-side stories, and 7.9% for pro-establishment respondents about their-side stories.

In the U.S., our Republican respondents answered that they had heard of the Republican-media stories 68.2% of the time, but our Democratic respondents answered that they had heard of the Republican-media stories only 38% of the time, *t*(201) = 7.0, *p* < 0.001, *d* = 0.98, 95% CI [0.70, 1.26]. Similarly, our Democratic respondents answered that they had heard of the Democratic-media stories 70.9% of the time, but our Republican respondents answered they had heard of the Democratic-media stories only 49.2% of the time, *t*(201) = 5.5, *p* < 0.001, *d* = −0.77, 95% CI [−1.05, −0.49]. Meanwhile, Democratic respondents answered that the Democratic-media stories were “common knowledge” 27.2% of the time versus 24.5% for Republican respondents, *t*(201) = 0.8, *p* = 0.449, *d* = −0.11, 95% CI [−0.38, 0.17], and Republican respondents answered that Republican-media stories were “common knowledge” 29.2% of the time versus 18.3% for Democratic respondents, *t*(201) = 3.3, *p* = 0.001, *d* = 0.46, 95% CI [0.18, 0.74]. These are similar percentages to the more epistemically sophisticated answer (“I heard about it, but I do not think many [of the opposing party] know about it”), which was selected 30.1% of the time by Democrats about Democratic-media stories, and 25.7% for Republicans about Republican-media stories.

The findings of Study 1 suggest that the CoK may be misleading some partisans to overestimate the extent to which the politically relevant information they know is widely shared. Our respondents in Hong Kong exhibited a greater degree of overestimation compared to our U.S. respondents, which may be an artifact of the particularly charged environment at the time. But in both contexts, either majorities or sizable minorities mistook their own knowledge of political news for common knowledge, when that knowledge was not actually shared in common. Democrats did not evince this overestimation, while Republicans did; but to a lesser degree than both groups of partisans in Hong Kong. Likewise, U.S. partisans were more likely to select the epistemically sophisticated option, acknowledging knowledge gaps between partisan groups – possibly the result of wider awareness of political polarization and media bias. However, simply overestimating the degree to which partisan knowledge is shared might not exacerbate polarization on its own. Contrariwise, the greater likelihood of selecting the epistemically sophisticated option in the U.S. might not reduce polarization, if such considerations do not come to mind in real-world contexts, without prompting. If the CoK contributes to polarization, such knowledge would be overestimated *and* political opponents would be judged more harshly on account of having this imputed knowledge, but persisting in their opposition regardless.

## 3. Study 2

Overestimating the extent to which politically relevant knowledge is widely shared would be of little consequence, if such overestimation did not result in harsher judgments of those whose actual knowledge leads them to take an opposing opinion. In this study, we explored whether receiving information about a new, fabricated political issue would lead toward harsher judgments of those disagreeing with the opinion such information would tend to support — despite being instructed that effectively no one else had been informed about it. We expected that treatment-group participants would make harsher judgments of opponents on the issue compared to those in the control group, overlooking the fact that their opponents had not received the same information.

### 3.1. Participants and design

Expecting a small effect size but without examples from the literature, we used G*Power to calculate the sample needed for a range of possible lower-end effect sizes; 1,048 participants were recruited via Prolific among self-identified Republicans and Democrats in the U.S.; 942 passed the attention check (a question testing factual memory of the treatment or control texts) and were included in the final analysis (487 women, 463 Republicans, *M*_age_ 37.6, SD_age_ 14.14).

### 3.2. Procedure and materials

Participants were randomly sorted into control and treatment groups. In the control, participants read a description of the executive branch of the federal government, focusing on the 15 federal executive departments. In the treatment group, participants read a fabricated announcement about a senior Department of Homeland Security official accused of accepting illegal bribes by a DHS whistleblower, who had just shared this accusation alongside incriminating evidence on the OpenSecrets website. The announcement noted further that the web page listing the accusation had received under 200 “hits” or visitors since it went public, and no media outlets had yet reported on the story, hence “it is safe to say that almost no one (beside you) has heard about it yet.” To ensure that participants read and understood the materials, they were given a multiple-choice question about the content, and were asked to briefly explain the reasons for their rating.

### 3.3. Measures

Participants were given an 11-point feeling thermometer to rate their feelings toward “those Americans who think that federal prosecutors should *not* focus more effort on investigating possible corruption in government agencies.” (Please see Appendix A for a discussion on the feeling thermometer, how it seemed to sometimes be misinterpreted, and what we did to clarify the interpretation of it.) A 0 represented “how you feel about your worst enemy,” and a 10 represented “how you feel toward the person you love most in the world.”

### 3.4. Results and discussion

We expected that participants in the treatment group would fail to account for the ignorance of those who might not see a need for federal prosecutors to divert their attention away from other concerns toward corruption in federal agencies, and judge people holding this opinion more harshly than those in the control group. We found that treatment-group participants did judge opponents on this issue more harshly (*M* = 2.27; SD = 2.03) than those in the control group (*M* = 2.53; SD = 1.93), *t*(942) = 2.0, *p* = 0.045, *d* = 0.13, 95% CI [0.01, 0.26].

This was a small difference, as would be expected in our theoretical model of how the CoK exacerbates polarization: We provided only a small piece of information at one point in time, whereas politically engaged partisans absorb large amounts of information over their lifetimes. As more information is learned cumulatively, the CoK would attribute more information to others who have not actually acquired it, making opposition to the opinions such information supports harder to explain other than by invidious motives.

However, this result might also be explained as an effect of priming: that treatment-group participants were primed to think of government corruption in general, and with this problem at the forefront of their minds, made less charitable judgments of those who disagreed that prosecutors should focus more on rooting out government corruption. In our next study, we investigated whether by focusing attention on what political opponents *do* and *do not* know about an issue, judgments of less knowledgeable opponents would be moderated.

## 4. Study 3

The CoK may contribute to polarization by obscuring differences in knowledge that resulted in differences of opinion. In this experiment, we tested whether judgments of political opponents would be moderated for those opponents who were described as being ignorant of the issue-relevant knowledge participants knew, compared to opponents who were described as sharing the same issue-relevant knowledge. We expected participants to judge political opponents less negatively if they were informed that they do *not* share the same knowledge of the issue — *if* this information overcomes potential CoK effects, and is interpreted to suggest that the opponent’s opinion may have been produced by the absence of issue-relevant knowledge known to participants — compared with participants who were informed that an opponent *did* share the same issue-relevant knowledge.

### 4.1. Participants and design

Without effect sizes from existing research, we tentatively expected a small effect size, as prior research has demonstrated the CoK to be robust against instructions to consider others’ knowledge or take others’ perspectives. We recruited 600 self-identified Republicans and Democrats in the U.S. via Prolific (295 women, 238 Republicans; *M*_age_ 39.21, SD_age_ 14.12). For a two-tailed *t*-test of mean differences with, this sample would have an 80% chance of finding a true effect of slightly over 0.2; but it would be underpowered to detect smaller effect sizes.

### 4.2. Procedure and materials

Participants were asked to name a political issue important to them, and to provide three facts they knew about the issue that support their opinion on it. Then they were instructed that text-mining software would search through notes from a previous interview-based study and match them with an interviewee to rate. Participants were randomly selected into two conditions. In both conditions, participants were presented with excerpts from interview notes; in the ignorant condition, the interview notes did not mention any of the facts the participant provided, and in the knowledgeable condition, the interview notes indicated that the interviewee did know the facts the participant wrote about. In both conditions, participants were informed that the interviewee expressed opposition to the opinion expressed by the participant.

### 4.3. Measures

Participants were given a 10-point scale with happy to angry faces as graphic references.

### 4.4. Results and discussion

No difference was found between the ratings of the interviewee who knew the same facts (*M* = 4.53; SD = 2.35) and one who did not know the same facts (*M* = 4.48; SD = 2.00), *t*(585) = 0.275, *p* = 0.784, *d* = 0.02, 95% CI [−0.14, 0.19]. Excluding participants whose stated issues and facts were independently judged as indicating inattention or misunderstanding by both authors did not affect results. This null result is consistent with the explanation that the CoK may *not* inflame polarization by harshening judgments of others via wrongly assuming them to know the same issue-relevant information. However, it is also consistent with previous research, which has established the robustness of CoK effects in the face of instructions to consider others’ knowledge and to take another’s perspective (Ryskin and Brown-Schmidt, 2014; Damen et al., 2018, 2020); here too, providing only evidence of what another knows and does not know did not affect judgments. Our next study sought to distinguish between these two explanations.

## 5. Study 4

Study 3 randomly provided either an example of a political opponent who knew, or did not know, the same issue-related facts as participants. In this study, we made knowledge gaps more visible by instructing participants to separately judge those who did and did not know the same issue-related facts. In this way, we hypothesized that if the CoK were harshening judgments of political opponents by occluding epistemology, participants asked to separately rate knowledgeable and ignorant opponents would be forced to grapple with political epistemology, considering how knowledge gaps might affect the development of an opposing opinion – and would judge less-informed opponents less harshly. We furthermore expected that ratings of knowledgeable opponents would be closer to the initial rating of all opponents, compared to ratings of ignorant opponents. That is, we expected participants to judge opponents who are *ignorant* of issue-relevant knowledge less harshly than opponents who were knowledgeable; and that ratings of knowledgeable opponents would be closer to ratings of opponents in general, evincing CoK bias. Alternatively, if the CoK were not influencing political judgments according to our theoretical expectations, ratings of more knowledgeable opponents might be the same or higher than ignorant opponents, owing simply to the positive quality of being knowledgeable.

### 5.1. Method

#### 5.1.1. Participants and design

Self-identified Republicans and Democrats in the U.S. (*N* = 152; without an expected effect size, funding limitations necessitated a small sample) were recruited through Prolific (70 women, 66 Republicans, *M*_age_ 35.34, SD_age_ 14.71).

#### 5.1.2. Procedure and materials

Participants were asked to name a political issue important to them and to list three relevant facts that back up their opinion on the issue. An example issue was provided: whether to create a new state park, along with three example facts that support a favorable opinion on the issue. After naming their issue and writing down three related facts, participants were asked to rate how they feel about people who disagree with them on this issue. In the next step, they were asked to separate those who disagree into two groups — first, opponents who know the facts they listed, and then those who do not — and to separately rate how they feel towards these two groups. Both authors independently examined the provided facts to verify good-faith effort and understanding of the instructions. Any differences in the coding were discussed and resolved. Excluding participants who failed these checks did not affect results, so all data are reported below.

#### 5.1.3. Measures

Participants were given 11-point scales with happy to angry faces as graphic references, with higher ratings indicating harsher judgments.

### 5.2. Results and discussion

Participants rated those who shared the same issue-relevant knowledge yet disagreed with their opinion (*M* = 7.92; SD = 2.40) significantly more negatively than those who disagreed with them but were unaware of the same facts (*M* = 5.72; SD = 2.21), *t*(151) = 10.846, *p* < 0.001, *d* = 0.88, 95% CI [0.69, 1.07]. When participants were directed to separately consider their feelings about those who *do* and *do not* know the same issue-related facts, they were more forgiving of opponents who lacked the knowledge participants deemed important to understanding the issue. The rating difference between all opponents and opponents who lacked the same knowledge (*M* = 1.82; SD = 2.55) was greater than the rating difference between all opponents and those who knew the same facts (*M* = –0.38; SD = 2.03), *t*(151) = −10.846, *p* < 0.001, *d* = −0.88, 95% CI [−1.07, −0.69]. This indicates that when people think of political opponents in general, they judge them in much the same way as they judge opponents who know what they know about an issue — a curse of knowledge effect (i.e., imputing one’s own knowledge to all others). When thinking separately about opponents who do not share the same knowledge, instead of punishing them for their ignorance, they were judged more charitably: opponents’ ignorance of the knowledge that supports one’s opinion was treated as a mitigating factor. The final study uses an experimental design to look for CoK effects by testing an attempt to debias the curse of knowledge.

### 5.3. Study 5

Study 3 found that providing information on what political opponents know or do not know about an issue did not affect judgments. But Study 4 found that by focusing attention solely on the difference between opponents who know the same facts as the participant — the facts that shaped their position on the issue — and opponents who were ignorant of those facts, participants rated the ignorant more favorably, and opponents who knew the same facts more harshly. To look for clear evidence of a CoK effect in political judgments of others, we designed a final experimental study testing an attempt to debias the CoK. If the CoK were influencing affective polarization – and such polarization were not exclusively caused by other factors – participants receiving a simple political epistemology explanation of why people may disagree should make more moderate judgments. We expected that judgments of political opponents who lacked participants’ issue-relevant knowledge would be moderated by an epistemological treatment instructing participants to consider how this ignorance may influence an opponent’s opinion. Alternatively, if the CoK were not negatively influencing judgments of political opponents, this attempt to debias a nonexistent influence should have no effect on judgments.

### 5.4. Participants and design

Tentatively expecting a mid-range effect size, 200 participants were recruited via Prolific among self-identified Republicans and Democrats in the U.S. (100 women, 102 Republicans; *M*_age_ 38.11, SD_age_ 14.75). This sample would have 0.95 power to detect a true effect size of at least 0.4 in a two-tailed *t*-test.

### 5.5. Procedure and materials

As in Studies 3 and 4, participants were asked to name a political issue of importance, and list three facts supporting their position on the issue, with the same example provided. They were instructed that text-mining software would search through notes from a previous interview-based study and match them with an interviewee to rate. Participants were randomly selected into treatment and control conditions. In the control, participants were presented with excerpts from interview notes, presenting “Jessica” as “very knowledgeable” in general, but “when we asked Jessica about <participant’s issue>, she did not seem to know as much about this issue as the other issues we discussed; she explained that this is an issue she has not yet learned much about.” The specific facts participants had written were presented, alongside a low “text-mining similarity score” of 5% indicating that “Jessica *does not know* the same facts that support your opinion, and she takes the opposite position on this issue.” The debiasing treatment condition was the same, except this information was followed by an explanation that political disagreements are sometimes caused by a lack of knowledge held in common — since what we know and do not know about an issue influences the opinion we develop — and other times by different value judgments. The example provided pre-treatment was then used to illustrate how sometimes learning more about an issue may change one’s opinion, but such new knowledge might also leave one’s opinion unchanged if it is rooted in conflicting values or beliefs, or differing interpretations of the same information.

### 5.6. Measures

Participants were asked to rate “Jessica” on a feeling thermometer, from 1 “Strongly dislike” to 10 “Strongly like.”

### 5.7. Results and discussion

We expected that the debiasing treatment would moderate judgments by reducing CoK effects, and found that participants in the debiasing treatment rated their political opponent more favorably (*M* = 5.22; SD = 2.08) than those in the control (*M* = 4.40; SD = 1.89), *t*(200) = 2.9, *p* = 0.004, *d* = 0.42, 95% CI [0.14, 0.69]. There was no significant difference in the results when eliminating validity check failures, so results from the full sample are reported.

If the CoK were not negatively influencing judgments of political opponents, the treatment focusing attention on the epistemology of political disagreement should have made little difference. Negative judgments based on a “know *or should know*” standard, not a CoK overestimation of knowledge, would unlikely be affected by this treatment. But here, as in Study 4, focusing participants’ attention on the role of knowledge and ignorance in the formation of political opinions – alongside the alternative possibility that differences in values and beliefs may make knowledge gaps irrelevant – resulted in more favorable, less harsh judgments of a political opponent. This provides additional evidence that the CoK, by occluding epistemology and exaggerating similarities in knowledge, makes an independent contribution to political polarization.

## 6. General discussion

If the curse of knowledge affects political cognition, one likely effect is exacerbating political polarization. Partisans would make the CoK error of unconsciously assuming that their political opponents know the same information that they themselves have learned, and which led them to form the opinion rejected by their opponent. With the possibility occluded that one’s opponent has not learned the key information that led to the formation of one’s own opinion, how is one to explain the opponent’s position? Ignorance aside, the remaining options — laziness, self-interest, ideological bias, malice — all paint the opponent in a negative light. This is less likely to occur regarding casual or ambivalent opinions, without much personal investment or about which the partisan merely leans to one of several known, well-supported sides. But for strongly held opinions, where “the other side” seems self-evidently wrong or immoral given what the partisan knows (and does not know), the CoK would tend to make opposing opinions unfathomable – except as motivated by discreditable intentions.

To investigate, Study 1 first collected evidence that partisans overestimate the extent to which news stories in their preferred media outlets were also known by their political opponents. This is a clear CoK effect, related to pluralistic ignorance and the false consensus effect, but on its own might not contribute to political polarization. If the CoK does tend toward worsening political polarization, partisans would gather information leading them to form an opinion on an issue, unthinkingly assume that such information is universally held, and then, blind to the fact that others might not have learned the same information, judge those with an opposing opinion more harshly. Study 2 provided evidence of this process: participants were given unique information about an issue, formed an opinion on it, and despite being told that most people have not learned the same information, tended to judge those with a differing opinion on that issue more harshly. Study 3 used a more ecologically valid design, asking participants to make judgments of someone they met online with an opposing opinion on an issue. With only this person’s level of knowledge about the issue experimentally manipulated, most participants did not evince counter-CoK thinking. For example, they did not take the other’s ignorance and knowledge into account and temper their judgment with the charitable interpretation that what the other does not know might prevent her from forming the same opinion. As in Damen et al.’s (2018) study, this indirect attempt to get participants to focus on another’s knowledge did not succeed. However, these results are also consistent with an absence of CoK effects in judging political opponents. Contrariwise, assuming that the information provided about opponents’ ignorance did reduce CoK bias but the true effect size was small, our study was underpowered to detect it.

Study 4 provided a more direct intervention, asking participants first to judge those who oppose them on an issue of personal importance, and then asking them to rate separately those who know the same factual information relevant to the issue, and those who are ignorant of such information. With political epistemology brought to the fore of their minds by separating opponents into those who do and do not share the participant’s issue-relevant knowledge, participants made more charitable judgments of their opponents who were unaware of the information shaping participants’ opinions. Meanwhile, their judgments of opponents who shared the same information were as hostile as the judgments they made before considering the role of knowledge gaps. In Study 5, a similarly externally valid setting as Study 3 was used: again, making judgments of strangers online based on their political opinions. While past attempts to mute CoK effects have proven largely ineffective, this attempt was at least partially successful. When instructed to consider the basics of political epistemology — that opinions are formed on the basis of the information one has acquired, plus values and beliefs which affect the interpretation of that evidence, such that some may arrive at opposing opinions simply because they do not know the same information — participants judged a political opponent, on a self-selected issue of personal importance, less harshly. Taken together, these studies indicate that the curse of knowledge is one of several psychological contributors to political polarization, and that engaging in epistemological thinking may reduce its effects.

### 6.1. Limitations

The limitations of these studies include recruiting using an online platform, which limits the sample to those with access to the internet and basic computer literacy. The samples included only political partisans, and were non-representative on several demographic categories; representative samples may reveal differences between demographic groups. We took ecological validity into account in designing our studies, e.g., by presenting a person who they might meet online. However, the example person’s characteristics could influence the results; providing a representative array of example people would have remedied this problem but was not feasible. Another limitation lies in depending on self-reported awareness of different news stories. Varying degrees of social desirability bias and humility in admitting what one does not know could have influenced the results. Studies 4 and 5 were limited by relatively small sample sizes. Our manipulation checks may have eliminated participants who were paying attention to the experimental materials, but whose attention lapsed only during the attention-check question, or whose written answers were incorrectly judged as indicating misunderstanding or inattention.

With regard to the possible confounding effect of priming in Study 2, future research could present the control with an old news story about the same treatment topic, i.e., an example of corruption, that received sufficiently ample media coverage as to be nearly universally known. In this way, the topic would be salient in both the treatment and control conditions, eliminating the potential priming effect. Another possibility would be to give the control condition the same story but tell the control group that everyone has heard of it, or omit information about who knows it. However, this design has interpretation difficulties: if the experimental group judged opponents less harshly than this control, it could be that the instructions alerting participants to the lack of media attention (only the participant is likely to have heard of it) debiased default CoK effects. At the same time, if there were no difference in ratings between the two groups, this could be the result of the experimental group instructions being overwhelmed by the CoK bias, as has occurred in prior research. In other words, experimental-group participants could have defaulted to CoK over-imputation of knowledge to others, making their ratings equivalent to those in the control group. Another possibility would be to use a three-group design: (1) the original experimental treatment, (2) a condition told that widespread media coverage means that nearly everyone has heard of it, and (3) a control with no information about who has heard of the allegations. All else being equal, those in the original experimental treatment would be expected to have the least harsh judgments of opponents, as their instructions should at least somewhat reduce CoK effects by focusing attention on widespread ignorance of the story. No difference would be expected between the group told that everyone has heard of it and the control group in which no information about others’ knowledge was provided. A manipulation check would be needed to ensure that those in the group which was told that essentially everyone had heard of the story, actually believed that there was widespread media coverage sufficient to ensure that effectively everyone would know of it.

For greater ecological validity, studies building on the successful debiasing procedure in Study 5 should try introducing participants to others whose level of knowledge is *not* stated, to explore how depolarization efforts can be best designed for most real-world situations in which political opponents’ knowledge and ignorance is unknown. Furthermore, our U.S. story selections relied on editorial decisions made by producers at the most popular cable opinion shows; a better method of selecting those stories of greatest interest to contrasting partisans may be to exploit engagement data from social media companies, where available. Future research could also try to exploit any existing measures of how often a news story is covered by media sources on one side versus the other. Lastly, the difference in the salience of polarized political debates during the 2019 Hong Kong protests and the U.S. of early 2022 may be a contributing cause of the lesser overestimation of contrapartisan knowledge in our U.S. data. Collecting similar data during a presidential campaign season in the U.S. may result in more similar levels of overestimation. Alternatively, the greater degree of collectivism in Hong Kong compared to the U.S. may have affected the likelihood of respondents to select the “common knowledge” response.

### 6.2. Theoretical implications

This study is the first to demonstrate that politics is another domain in which the CoK affects cognition, and provides evidence that its effect is to exacerbate political polarization. Political polarization has been increasing over recent decades, but psychological biases, the CoK included, have likely remained unchanged over the period. A constant being unable to explain a variable, clearly the CoK cannot be *the* cause of increasing political polarization in many countries. Rather, the CoK is likely an adjunct or accelerant to the central causes of increasing political polarization.

For instance, changes in the U.S. media system are a central cause of political polarization there (Prior, 2007). Before the rise of cable and then the internet, broadcast television news was more widely watched and influential; and to attract the largest possible audience, political news tended to be presented in a down-the-middle, nonpartisan manner. The introduction of cable television vastly expanded the number of options for viewers, and helped create a niche for news channels with a decidedly partisan bent. Buoyed by the market success of partisan news outlets, and with social media algorithms facilitating ideologically homogeneous communication networks, the U.S. media system became more populated with content designed to appeal to opposing partisan groups (Taibbi, 2020). With separate media diets providing contrasting perspectives on political issues, as well as covering different stories entirely (e.g., Radtke, 2017), not only were partisans entitled to their own opinions — they were presented with their own sets of facts. As partisan groups accumulate differing sets of politically relevant knowledge, they become more susceptible to CoK effects. For instance, Republicans absorbing copious information from their partisan media diets about problems attributed to immigration would wonder why Democrats seem unconcerned about a problem they have learned has caused tremendous suffering to U.S. citizens. Blinded by the CoK to the explanation that Democrats’ media diets do not include so many stories about victims of immigrant criminals and public services overwhelmed by newly arrived migrants, other explanations must be found (e.g., “Democrats tend to be more privileged, do not face these problems in their own lives, and so do not care about working class Americans who have to live where such problems are most acute”). Democrats absorbing information from their partisan media diets about the existential threat of climate change would wonder why Republicans seem so unconcerned about it. Blinded by the CoK to the explanation that Republicans’ media diets do not include so many stories explaining the danger of climate change or linking destructive weather events to it, other explanations must be found (e.g., “Republicans are anti-science, and they care more for oil companies than life on earth”).

Whereas in economic contexts the CoK may produce socially beneficial effects by making information asymmetries more difficult to exploit, in political contexts, as in many others, the CoK is more likely to contribute to social harms, like an increasingly polarized society.

### 6.3. Practical implications

If the curse of knowledge is merely an accelerant or partial cause of political polarization, then muting its effects is unlikely to solve the problem entirely, but it would ameliorate it. The results of Study 5 suggest that thinking about political epistemology, if not eliminating the CoK, may reduce its negative effects on judging one’s political opponents. Political epistemology, or how people come to know or believe what they know or believe about politics, involves many factors: what one learned from one’s parents, peers, teachers, media diet, life experiences, and other sources of politically relevant information, along with psychological traits that draw one toward some ideas and away from others (Beattie, 2019). By drawing attention to the fundamental arbitrariness of the process by which we accumulated some knowledge and not other knowledge, and realizing that what we have and have not learned affected the development of our political opinions, we may be humbled (if not humiliated). But so too are our political opponents: their opinions were also formed through a fundamentally arbitrary process of learning some things we likely have not, and not learning other things we have. Focusing the attention of partisan disputants on knowledge gaps between them may make attributions to malice less likely, and attributions to ignorance more likely. And if the apparent solution to an opponent motivated by malice is combat, the solution to opposition rooted in ignorance should be dialogue.

In commercialized media systems, where media outlets compete for advertisers and subscribers, educating audiences in political epistemology is unlikely to occur unless such efforts result in greater revenues. If audiences reward such efforts, they would likely spread across the media system; but if audiences prefer partisan animosity from their media diets, political epistemology is unlikely to be featured. However, educators could teach basic political epistemology alongside media literacy in schools. Students would be taught to critically analyze the news media, considering (among others) potential sources of bias and the adequacy of evidence provided to support an argument or explanation, and also to think about how what they and others learn (and do not learn) influences opinion formation. For such educational interventions to succeed at reducing polarization — or at least the portion of polarization produced by the curse of knowledge — additional research is needed.

## 7. Conclusion

The present studies extend research on the curse of knowledge to the domain of political cognition, by demonstrating that overestimating political opponents’ knowledge is linked to more negative appraisals. When partisans commit the CoK error of assuming that political opponents share the same knowledge as themselves, opponents take on the malevolent character of one who knows why a differing opinion is correct, yet persists in opposing it.

This theoretical understanding led us to develop and successfully test an intervention to debias CoK effects: prompting partisans to think like political epistemologists. By engaging in thinking about how differences in knowledge affect opinion formation, partisans may find their opponents less implacable, and their character less that of an enemy and more that of one who could be made an ally through dialogue. Indeed, the opponent must always be explained; but if the *first* explanation that we look for is that he sees a different set of facts, political polarization may be reduced.

## Data availability statement

The datasets presented in this study can be found in online repositories. The names of the repository/repositories and accession number(s) can be found at: https://osf.io/yc3tf/?view_only=525a070de6a74410aaa445f3f97cbbec.

## Ethics statement

The studies involving human participants were reviewed and approved by Survey and Behavioural Research Ethics, CUHK. The patients/participants provided their written informed consent to participate in this study.

## Author contributions

PB did the original research in Hong Kong and the initial literature review. PB and MB collaborated on theoretical development, research design, and data analysis and wrote and revised the manuscript. MB performed the additional literature review. All authors contributed to the article and approved the submitted version.

## Funding

This research was funded by a generous grant from CUHK’s MGPE Research Fund.

## Conflict of interest

The authors declare that the research was conducted in the absence of any commercial or financial relationships that could be construed as a potential conflict of interest.

## Publisher’s note

All claims expressed in this article are solely those of the authors and do not necessarily represent those of their affiliated organizations, or those of the publisher, the editors and the reviewers. Any product that may be evaluated in this article, or claim that may be made by its manufacturer, is not guaranteed or endorsed by the publisher.
