# Supplementary material for: Political polarization: a curse of knowledge?
Source: Front Psychol. 2023 Jul 12;14:1200627. doi: 10.3389/fpsyg.2023.1200627 (PMC10368969; doi:10.3389/fpsyg.2023.1200627)
Supplement: Supplementary file 1 [file Table_1.DOCX]

**Appendix A**

In this appendix, we provide a brief narration of our pilot testing to be transparent and in case our experience may be of utility for researchers.

**Study 1**

As described in the article, we were first alerted to the possibility that the curse of knowledge might affect polarization during the 2019 protests in Hong Kong. One of our social media feeds had roughly equal numbers of “blue” and “yellow” supporters, which made apparent how partisans in this more-recently polarized society were sharing and discussing entirely different stories and events relating to the protest. Plans to extend initial research carried out during the protests had to be canceled, as the random-digit dialing survey company that had agreed to carry out a representative survey canceled due to covid restrictions affecting their office. We began reviewing the literature on the curse of knowledge, and decided to engage with this research project focusing on the U.S. context in late 2021 after receiving funding.

First, we replicated the same study on knowledge of events in the news in the United States, finding that partisans there similarly tended to overestimate the extent to which news stories in their own media diets would be widely known outside of their partisan group. Evaluations between partisan groups were done by adding those who selected one of the answers indicating knowledge of the story, and comparing them with those who selected the answer indicating ignorance of the story. Separately, percentages of those selecting the “common knowledge” option were compared to those selecting one of the more epistemically sophisticated responses.

**Study 2**

Next, we designed experiments to test a link between overestimating opposing partisans’ knowledge and more negative evaluations of one’s political opponents (as if they *should* take one’s own position, since they are assumed to know the evidentiary basis for one’s own position, and hence their opposition is a moral or intellectual failure). Our first pilot test used the hot-button issue of immigration, showing Republican participants an article on a new (fabricated) legislative proposal to crack down on illegal immigration by prosecuting business that hire undocumented immigrants, and Democratic participants an article on a new (fabricated) legislative proposal to address the climate crisis by hiring fossil fuel workers to install and maintain renewable energy infrastructure. After reading the articles (or a control article on an apolitical topic), we asked them to rate their feelings for those who opposed the proposals. We included an open-response question asking participants for their reasons in rating the opponent the way they did, and from these we discovered that Republican and Democratic participants did not uniformly agree with the respective proposals, which was essential for the study. Another concern we had was that immigration (for Republicans) and climate change (for Democrats) were such top-ranked issues that learning additional information about them would not produce the expected measurable effect: information about these proposals would be added to a large, previously existing store of issue-relevant knowledge, such that the new knowledge may have a negligible effect on ratings of one’s opponents.

Our next pilot study selected a relatively more recent issue, police brutality, which in recent years had emerged as a major focus of partisan dispute. Although we perceived the issue to be relatively newer for U.S. partisans compared to immigration and climate change, the open-ended answers we received indicated that the sampled Republicans and Democrats did not uniformly agree with the partisan narratives we included in the treatment articles: for Republicans, that disrespect for police and calls to defund them were leading to more violence against police officers, and for Democrats, that police violence against minorities call for serious legislative reforms. Ratings of the opponent seemed to be powerfully influenced by pre-existing beliefs and knowledge related to the issue, with our treatment articles associated with minimal effects on opponent ratings.

According to our theoretical understanding of how the curse of knowledge might increase polarization, issue-relevant knowledge would be accumulated over time, and each additional bit of information would be associated with only a small effect on feelings toward one’s opponents on the issue. For instance, a Republican who initially knew little about immigration as a political issue would begin to accumulate information about it through their media diets and social networks - stories of undocumented immigrants who commit crimes, unemployed citizens who are rejected by employers in favor of cheaper immigrant labor, diseases purportedly carried by migrants, etc. - and as each bit of information that would support an anti-immigration opinion is absorbed, and it is unthinkingly assumed that everyone else has learned the same information, one’s opinion of an opponent on this issue would deteriorate progressively. (“After all, *everyone* knows all of these facts that have led me to take an anti-immigration position, so my opponent must simply not care about all the victims of immigrant criminals I’ve heard about, or the countless citizens I have heard from who suffer unemployment because jobs are going to immigrants, etc.”) Hence to capture this effect at one point in time, we decided to create an entirely fabricated political issue: how to potentially respond to aliens. We thought we could succeed in providing new information better by introducing a novel issue that has not been part of public debate. However, we made a mistake by assuming Republicans would want to respond militarily and Democrats diplomatically. Republicans actually rated Democrats who supported a diplomatic response more positively (while our hypothesized result would have been a more negative rating and our null hypothesis would be that there would be no difference). Therefore, we tried to think of an issue that is non-partisan, i.e., that everyone would theoretically see as a problem: corruption. We also made sure that the allegations of corruption would not be associated with a Republican or Democratic perpetrator, again to try to make the information non-partisan. This last story about corruption in the Department of Homeland Security, which we used for both Republicans and Democrats, is reported in the article.

**Studies 3-5**

Since the design of Study 2 could be interpreted as producing priming effects, not just new-knowledge effects, we wanted to follow it up with a study with similar external validity - measuring feelings toward political opponents one might meet online - but which could not be interpreted as producing a priming effect. Our first pilot test asked for ratings of a Trump supporter and opponent, and a pro-life and pro-choice person, from Republicans and Democrats, respectively. The information provided about the opposing-party ratee was limited to fabricated results of a knowledge test about Trump (with facts that paint him in a positive light for Republicans, and a negative light for Democrats) and abortion (similarly, with facts that are used by pro-lifers for Republicans, and facts used in pro-choice arguments for Democrats). In the “knowledgeable” condition, the ratee answered all five questions correctly (evincing a grasp of knowledge fellow partisans would be more likely to know), and in the “ignorant” condition, the ratee answered all five questions incorrectly. Past research on the curse of knowledge suggested that it is difficult to debias or correct, but we thought that by presenting only information on the knowledge or ignorance of one’s opponent would focus participants’ attention on matters of political epistemology, and possibly succeed at debiasing: leading participants to rate Democratic (Republican) opponents who knew favorable (disfavorable) facts about Trump, and facts used in pro-life (pro-choice) arguments, more harshly than Democratic (Republican) opponents who were ignorant of these facts that support the rater’s own position on these two issues. As a final test, we informed participants that a software bug had shown the wrong test results initially, and those in the knowledgeable condition were shown the ignorant condition presentation on abortion, and vice versa. However, pilot testing found no overall effect of knowledgeable and ignorant conditions, before or after the “correction”; and open-ended responses revealed some people thinking as epistemological realists (e.g., because the ratee seems ignorant of the facts supporting my opinion of the issue, I cannot rate her harshly; she does not *reject* my position so much as *not know* the reasons why I take my position), and many more thinking as naive realists (e.g., I rated her harshly because she is so ignorant about an important issue).

But as with previous attempts in the research to debias the curse of knowledge, simply highlighting what ratees did or did not know about an issue did not spur the majority of pilot participants to engage in political epistemology, or consider the implications of what knowledge opponents have and do not have, judging by responses to the open-ended question. Hence in Studies 4 and 5, we took a more direct approach, as reported in the article.

We decided to ask participants to give their own facts to make the studies more realistic and relevant. In the studies which asked participants to give facts, both authors checked to make sure the participants gave facts — i.e., not opinions or the copied-and-pasted example, but actual, participant-provided facts that would make sense when they are told someone does not know them — and resolved any differences in opinion. We disregarded the veracity of the facts as it was not relevant to our study. We also had open answer responses to find out more about what the participants were thinking, e.g., when rating people or about the survey design. In the third study, we had an attention check, both authors checked the facts to see if they were facts (true or untrue) rather than arguments/opinions and if they could conceivably too common to be unknown to someone else, a manipulation check, and a check to see if the participants thought the test mining was fake. We also asked why the participants rated the target as they did. In Study 4, the open answers informed us when a Qualtrics scale was not user friendly so we changed the scale type for future participants. In Study 5, we again asked why the participants rated the target the way they did and gave them a manipulation check and a check to see if the text mining seemed fake.

An unexpected finding we had was many participants focusing on blaming the target for being ignorant, so we revised the description to try to make both conditions’ targets seem equally knowledgeable. We changed the descriptions to say that the target is “very knowledgeable in general”, or that she listed other facts on the issue, but she just doesn’t know those specific facts that the participant listed.

In using the feeling thermometer, we noticed that there seemed to be misinterpretations. Sometimes the target person or group was rated as warm, but in the open answer they said that they thought negatively about the person or group. We think that the thermometer might be interpreted the opposite as it should if, e.g., the participant sees it as measuring anger (like is sometimes illustrated in cartoons) and does not read the accompanying text carefully. Then higher scores mean more anger. We ended up using a Likert scale with smiley faces ranging from happy to angry and explained the rating scale with four descriptors: negative/positive, less friendly/more friendly feelings, worst first impression/best first impression, and strongly dislike to strongly like on the scale itself. In this way, we tried to be as clear as possible and did not see the same contradicting open answers in future batches.
